# Multiple objective optimization of air assisted liquid-liquid microextraction combined with solidified floating organic drop microextraction for simultaneous determination of trace copper and nickel

**DOI:** 10.3906/kim-2010-24

**Published:** 2021-03-10

**Authors:** Fereshteh ISLAMI-BONAB, Sanaz SAJEDI-AMIN, Saeed Mohammad SOROURADDIN, Simin MALEKNIA, Abdolhossein NASERI

**Affiliations:** 1 Department of Analytical Chemistry, Faculty of Chemistry, University of Tabriz, Tabriz, Iran; 2 School of Mathematical and Physical Sciences, University of Technology Sydney, Sydney, Australia

**Keywords:** Air assisted liquid-liquid microextraction, green chemistry, heavy metals, natural waters, solidified floating organic drop

## Abstract

The impact of rising levels of various heavy metals in the environment from multiple industrial, agriculture, domestic, and technological activities is of great concerns, as heavy metals cause serious health effects for both humans and wildlife. An effective and green chemistry procedure was used for simultaneous extraction of ultra-trace copper and nickel ions in natural waters (e.g. river and well waters) by air assisted liquid-liquid microextraction combined with solidified floating organic drop microextraction (AALLME-SFOD). The extraction of metal ions was conducted in a timescale of several minutes by conversion to metal chelates using sodium diethyldithiocarbamate (DDTC) as a chelating agent prior to AALLME-SFOD procedure, and 1-dodecanol as an extraction solvent to initiate the phase separation of the complex. The response surface methodology (RSM) and central composite design (CCD) were used to optimize the large number of experimental variables (i.e. pH, solvent volume, ligand volume, % NaCl). The analysis of variance (ANOVA) has been applied to evaluate all parameters and their mutual interactions on the extraction yields. The desirability function of Derringer and Suich was applied as a metric for optimization of multiple response variables. The optimized AALLME-SFOD technique proved to be highly effective for the pre-concentration of Cu(II) and Ni(II) from a range of aqueous media (i.e. river and well waters) and provides a quick and easy method, while utilizing compact and low cost equipment with micro-volume organic solvent consumption (e.g. ~120 μL for 5 mL water samples). Quantitation of copper and nickel with Graphite furnace atomic absorption spectroscopy (GFAAS) under the optimum conditions, were linear over the range of (20–100) and (20–200) ng L^−1^ respectively. Limit of detection for Ni(II) was at 4.5 ng L^−1^ and 10.4 ng L^−1^ for Cu(II).

## 1. Introduction

The rising levels of various heavy metals in the environment from a range of human activities, especially in aquatic environments, is of great concerns as heavy metals cause serious health effects for both humans and wildlife. Major efforts of environmental scientists focus on quick and effective methods for detecting and analyzing both the types and trace levels of heavy metals in various matrices [1–6]. The preconcentration and determination of metal ions in aqueous solutions have been evaluated by several techniques such as solid phase extraction (SPE), liquid-liquid extraction (LLE) and micelle mediated cloud point extraction (CPE) [7,8]. In particular, development of microextraction (ME) techniques for the analysis and preconcentration of heavy metals in natural waters (e.g. rivers and wells), along with green chemistry infrastructure are of interest [1,9].

Optimization of sample preparation is regarded as the most significant primary step in an analytical procedure, and is critical for very low concentration of target analytes (i.e. parts per million (ppm) to trillion (ppt)) in complex matrices [10]. Since 1990s microextration (ME) has been an integral part of sample treatment, offering scientists a more effective, and relatively simple procedure with a minimum volume of organic solvents [11]. ME is divided into two categories due to the types of the extraction media as solid phase microextraction (SPME) [12,13] and liquid-liquid microextraction (LLME) [14–16].

The most important advances in LLME began in 2006 with the introduction of dispersive liquid–liquid microextraction (DLLME) technique [17]. This method is based on separating or partitioning compounds or metal complexes by their relative solubility in two different immiscible liquids (e.g. water (polar) and an organic solvent (nonpolar)). The main drawback of LLME is due to the use of chlorinated solvents [18,19]. The solidified floating organic drop microextraction (SFOD) was developed as an attempt toward greener extraction methods [20]. Despite the widespread interest of DLLME providing simplicity, fast performance with little solvent consumption, it is less efficient due to low contact area with the organic droplet. These shortcomings were resolved with the use of a disperser solvent, resulting in the development of DLLME-SFOD [10,21, 22], and further by introducing ultrasound emulsification microextraction and air agitation as efficient and preferable dispersion techniques, USAEME-SFOD [23–25] and AALLME-SFOD [26–28], respectively.

**Scheme 1:**
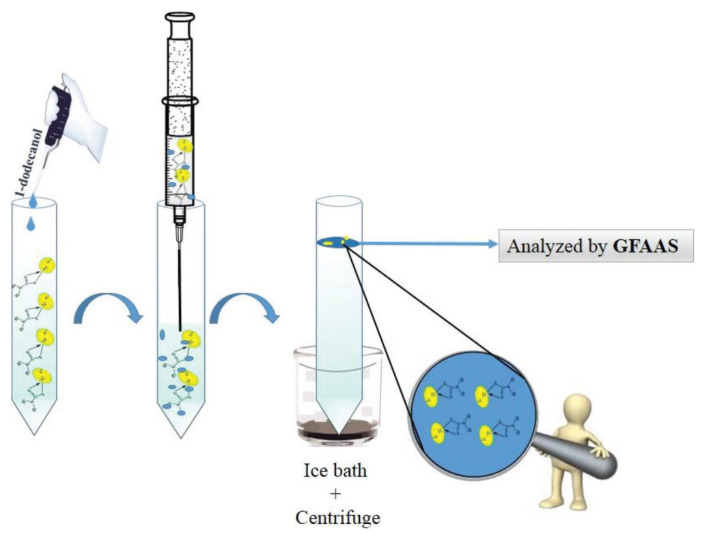
The AALLME-SFOD procedure through microextraction of Cu(II) and Ni(II).

The AALLME technique works on the basis of withdrawing the sample mixture by a syringe and transferring it out into a tube in a particular sequence as shown in Scheme 1. In this integrally rapid method, no dispersant is used. In AALLME-SFOD, as its name suggests, organic solvents are replaced with less toxic, low density, and proper melting point organic solvents. AALLME-SFOD combines the advantages of both AALLME and SFOD and advances both techniques [19–21].

The ME methods have been coupled to various analytical instruments for the identification and quantitation of heavy metals. The most commonly used techniques include flame and graphic furnace atomic absorption spectroscopy (FAAS, GFAAS) [29–33], inductively-coupled plasma optical emission spectrometry (ICP-OES), and ICP mass spectrometry (ICP-MS) [34].

Simple metal ions do not extract into organic solvents and require a chelating reagent to convert the metal ions to ligand-metal complexes with a higher affinity to partition in organic media, prior to the ME technique. The present study reports optimized AALLME-SFOD for the determination of copper and nickel in natural water samples (e.g. rivers and wells) by GFAAS. Sodium diethyldithiocarbamate (DDTC) was used as a reagent to convert Cu and Ni to metal complexes. The experimental conditions affecting the simultaneous extraction of copper and nickel included, amount of chelating agent, volume of extraction solvent, pH, extraction numbers, sample volume, salting-out effect, centrifuge rate, and time. Due to the large number of variables, the experimental variables were optimized by the central composite design (CCD) combined with response surface methodology (RSM) according to the desirability function (DF).

## 2. Materials and methods

### 2.1. Source of water samples

Natural water samples included well water, river, and surface waters. Well water sample was collected from a farm in Bostanabad, located ~55 km south east of Tabriz, Iran. River water was collected in the spring season from Mehranroud river at approximately 5 km from central Tabriz, Iran. Surface water sample was from a pool on campus of University of Tabriz in the spring season. The water samples were collected in polyethylene (PET) bottles and then stored at +4 °C until analysis time.

### 2.2. Chemicals and reagents

A mixed stock solution of Cu(II), and Ni(II) (500 μg mL^−1^) was prepared by dissolving appropriate amounts of Cu(NO_3_)_2_·3H_2_O,and Ni(NO_3_)_2_·6H_2_O (all from Merck, Darmstadt, Germany) in deionized water (Ghazi company, Tabriz, Iran), and acidified using nitric acid > 1% to avoid precipitation. The stock solution was serially diluted to prepare the working solutions. Sodium diethyldithiocarbamate (DDTC), as a chelating agent, was obtained from Fluka (Buchs, Switzerland). 1-dodecanol, sodium chloride, hydrochloric acid, and sodium hydroxide were from Merck (Darmstadt, Germany).

### 2.3. Graphite furnace atomic absorption spectroscopy

Graphite furnace atomic absorption spectroscopy (Shimadzu 6300, Kyoto, Japan) offers high sensitivity and repeatability. In order to obtain the best possible analytical signal, optimization of furnace temperature program drying, ashing, and atomization temperatures were performed. The optimum conditions are listed in Table 1.

### 2.4. AALLME-SFOD procedure

A 5.0 mL of the working solution or water sample containing 4.5% (w/v) NaCl at pH 8.5 was poured into a 10 mL tube followed by a 140 μL portion of 0.05 mol L^−1^ DDTC (chelating agent) and the injection of 120 μL 1-dodecanol as the extraction solvent. Air-agitation cycles (10 times, sucking and injection by using a 5-mL glass syringe) were used to reinforce the extraction of the target analytes. The solution became cloudy after the air agitation cycles, and was centrifuged for 4 min at 6000 rpm. The sample tubes were kept in an ice bath to solidify the droplets, which were then easily removed and allowed to melt for the analysis. 10 μL of the melted sediment phase was analyzed by GFAAS. The procedure is summarized in Scheme 1.

The efficiency of the procedure was evaluated by the percent extraction recovery (%ER) and enrichment factor (EF) described by Equations 1 and 2.


(1) $$\text{EF}=\frac{{\text{C}}_{\text{Final phase}}}{{\text{C}}_{\text{initial phase}}}$$

(2) $$\text{ER}=\frac{{\text{n}}_{\text{final phase}}}{{\text{n}}_{\text{initial phase}}}\times 100=\frac{{\text{C}}_{\text{final phase}}}{{\text{C}}_{\text{initial phase}}}\times \frac{{\text{V}}_{\text{final phase}}}{{\text{V}}_{\text{initial phase}}}\times 100=\text{EF}\times \frac{{\text{V}}_{\text{final phase}}}{{\text{V}}_{\text{initial phase}}}\times 100$$

Where, C_initial_ is the concentration of analytes in the initial solution, and C_final_ the final concentration, which is calculated from a calibration curve. V_final_ and V_initial_ are the volumes of sedimented phase and aqueous solution, respectively.

### 2.5. Experimental design methodology

Analytical procedures often require optimizing multiple responses. Different strategies have been proposed while desirability function approach is one of the most popular choices in experimental optimization of multiple response processes [35–38]. This method is based on applying a desirability function for each individual response, d_i_. The desirability values range from 0 to 1, where a value of 1 represents full desirability and a value of 0 corresponds to complete undesirable response. The weighted geometric mean of the individual desirability (d_i_) is generated according the following Equation:


(3) $$\text{D}=\sqrt[{m}]{d_{1}d_{2}\;\ldots \;d_{m}}$$

Where, m, is number of responses considered in the optimization process. For the purpose of maximizing the response, the individual desirability (d_i_) is defined as represented in Equation 4:


(4) $$d_{i}=\{\begin{array}{ll} \;\;\;\;\;\;\;0 & if\;y_{i}<L_{i}\\ {\left(\frac{y_{i}-L_{i}}{U_{i}-L_{i}}\right)}^{s} & if\;L_{i}\leq y_{i}\leq U_{i}\\ \;\;\;\;\;\;\;1 & if\;y_{i}\geq U_{i} \end{array}$$

Where, L and U, are the lower and upper acceptable values to the response, respectively. Also s, is the weight. Thus, when s = 1, the desirability function is linear. Desirability functions provide impartial, efficient, and low-cost optimization of multiple response procedures.

AALLME-SFOD method optimization was achieved by central composite design analysis (CCD) using Minitab Statistical Software (version 14). Four independent variables (ligand volume (X_1_), % NaCl (X_2_), pH (X_3_), and solvent volume (X_4_)) according to the conditions presented in Table 2 were performed and analyzed by RSM to depict the main effect of the variables and their interaction with each other. Overall 29 experimental runs with 5 replicates in center point were made by CCD (See Table 3).

The second order polynomial model in (Eq.3) was applied to correlate the dependent and independent variables:


(5) $$Y=b_{0}+\sum_{i=1}^{4}b_{i}x_{i}+\sum_{i=1}^{4}b_{ii}x_{i}^{2}+\sum_{i=1}^{3}\sum_{j=i+1}^{4}b_{ij}x_{i}x_{j}$$

Where Y is the predicted response (Extraction recovery); x_i_ the independent variables, b_0_ is a constant or intercept; b_i_ the linear coefficient; b_ii_ the quadratic coefficients and b_ij_ the interaction coefficients. The significance of model was evaluated by the R^2^ and the lack of fit.

### 2.6. Desirability function

The Derringer–Suich function or desirability function was applied to obtain a maximum extraction recovery for Ni(II) and Cu(II) simultaneously. The values of the individual recoveries (d_Ni_, d_Cu_), which have a different range scaling, can be combined. Finally, a global D-value was calculated as the geometric mean according the following equation:


(6) $$\text{D}=\sqrt[{2}]{d_{Ni}d_{Cu}}$$

Desirability functions provide impartial, efficient, and low-cost optimization of multiple response procedures [30].

## 3. Results and discussion

In this study sodium diethyldithiocarbamate (DDTC) was incorporated with trace levels of heavy metals present in water samples to form metal complexes prior to the AALLME-SFOD procedure. DDTC is a water insoluble collector which can be extracted by a variety of microvolume organic solvents for simultaneous determination of Cu(II) and Ni(II). The ligand binds to metals such as Cu(II) and Ni(II) through its two coordination sites [39].

The final separation conditions were selected through the optimization process. A wide range of AALLME-SFOD parameters were optimized. Sample solution volume, number of extractions, and centrifuging rate and time were recommended initially, then with selected complex agent and extraction solvents other parameters were optimized according to CCD followed by the desirability functions described below for the various experimental procedures.

### 3.1. Optimization of extraction process

The optimum conditions have been evaluated by experimental design to result minimum time, reagents and experimental runs. The primary parameters affecting the AALLME-SFOD extraction efficiency such as sample solution volume, numbers of extraction, and centrifuging rate and time were investigated and optimized one variable at a time. The effects of other variables such as pH, solvent volume, ligand volume, %NaCl, and their mutual interactions on the extraction efficiency were studied by CCD.

#### 3.1.1. Optimization of numbers of extraction

**Figure 1:**
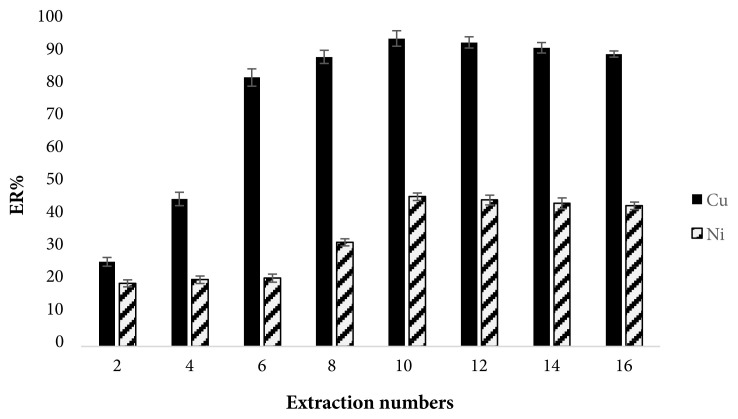
Effect of extraction numbers on ERs of Cu(II) and Ni(II). Extraction conditions: extraction solvent volume, 75μL ; sample, 5 mL deionized water containing 25 ng L^−1^ of Cu(II) and Ni(II) at pH 7 with NaCl, 1.5 % (w/v); SDDTC at 0.05 mol. L^−1^; centrifuge rate, 6000 rpm; and centrifuge time, 4 min. The error bars represent standard deviations (n = 3).

In this study, the numbers of extraction were the numbers of suction and injection of the mixture (sample solution + extraction solvent). The extraction equilibrium was obtained very quickly with increasing extraction numbers, which resulted the increased recoveries. The number of extractions was in the range of 2–16 times. As a result (Figure 1), by increasing extraction numbers, ER is also increased until the tenth extraction and then remains almost constant. Therefore, the optimal numbers of extraction was 10 times.

#### 3.1.2. Effect of sample solution volume

**Figure 2:**
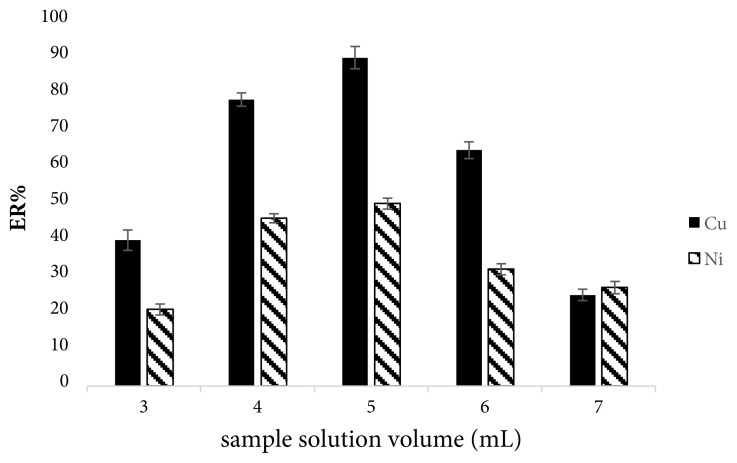
Effect of sample solution volume on ERs of Cu(II) and Ni(II). Extraction conditions: extraction numbers, 10 times; other conditions same as Figure 1. The error bars represent standard deviations (n = 3).

In order to obtain desirable extraction efficiency, sample solution volume was studied in the range of 3–7 mL. The increase in a sample size would generally result an increase in ER up to a point after which the increase would result an adverse effect on the formation of the organic phase drops. Based on the results presented in Figure 2, analytical signals increased by increasing the sample volume up to 5 mL and then decreased at higher volumes. This change occurs based on the relation between the extraction recovery and sample volume in Equation 2. When the volume of solvent increases for specific concentration of analytes, the amount of dissolved cations increase, the final concentration of copper and nickel ions in organic drop (C_final_) increases, which will cause an increase in ER%. On the other hands, adverse effect on ER% is observed with increasing sample volume due to the saturation of organic phase with analytes.

#### 3.1.3. Optimization of centrifuging rate and time

The effect of centrifuging rate and time on the extraction efficiency were studied within a range of 2000–10,000 pm and 2–8 min, respectively. The ER% Cu(II) and Ni(II) was found quantitative at 6000 rpm centrifugation for 4 min.

#### 3.1.4. Central composite design (CCD)

The 4 factors CCD matrix was designed for the extraction of copper and nickel from aqueous samples, which is provided in Table 2. Twenty-nine experimental runs were applied for the CCD, some of the runs were defined as outliers using studentized residual in the MLR model and were excluded. As shown, extraction recovery varied from 4.00%–78.38% for Ni(II) extraction, and 12.61%–100.00% for Cu(II) extraction. To evaluate the fitness, significance of the model for this study, analysis of variance (ANOVA) has been applied to find the effects of each variable and their mutual interaction on the response. Regression coefficients and analysis of variance of the second-order polynomial models for responses are summarized in Table 4. A p-value of less than 0.05 in the ANOVA table indicates the statistical significance at 95% confidence level. Based on these results the best fitted relationship between the response (ER % for Cu(II) and Ni(II)) and independent uncoded variables (X_1_–X_4_) are expressed by the following second-order polynomial Equations 7 and 8:


(7) $$\begin{array}{l} \;\;\;\%{\text{ER}}_{\text{Cu}}=-3.491\;{\text{X}}_{1}\;(\text{ligand volume})-35.0877\;{\text{X}}_{2}\;(\text{salt}\%)+22.4049\;{\text{X}}_{3}\;(\text{pH})+4.7783\;{\text{X}}_{4}\;(\text{solvent volume})-3.4838\\ {\text{X}}_{2}{\text{X}}_{2}-1.7855\;{\text{X}}_{3}{\text{X}}_{3}-0.0268\;{\text{X}}_{4}{\text{X}}_{4}+0.2751\;{\text{X}}_{1}{\text{X}}_{2}+0.017\;{\text{X}}_{1}{\text{X}}_{4}+3.9186\;{\text{X}}_{2}{\text{X}}_{3}-0.1541\;{\text{X}}_{3}{\text{X}}_{4} \end{array}$$

(8) $$\begin{array}{l} \;\;\;\%{\text{ER}}_{\text{Ni}}=395.916-2.837\;{\text{X}}_{1}\;(\text{ligand volume})-21.59\;{\text{X}}_{3}\;(\text{pH})-3.481\;{\text{X}}_{4}\;(\text{solvent volume})+0.005\;{\text{X}}_{1}{\text{X}}_{1}-0.664\;{\text{X}}_{3}{\text{X}}_{3}+\\ 0.004\;{\text{X}}_{4}{\text{X}}_{4}+0.112\;{\text{X}}_{1}{\text{X}}_{3}+0.015\;{\text{X}}_{1}{\text{X}}_{4}+0.939\;{\text{X}}_{2}{\text{X}}_{3}+0.09\;{\text{X}}_{2}{\text{X}}_{4}+0.188\;{\text{X}}_{3}{\text{X}}_{4} \end{array}$$

The model adequacies were checked by the determination of correlation coefficients (R^2^) and adjusted R^2^. The high values of the R^2^ and R^2^_adj_ and nonsignificance of “Lack of Fit” specify high predictability of the model.

**Figure 3:**
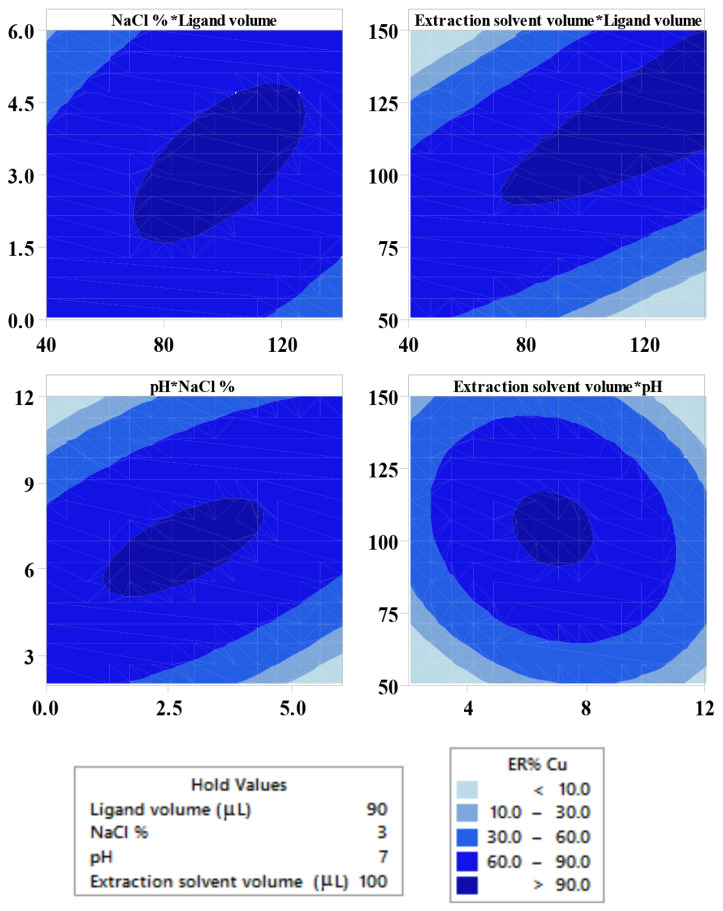
Contour plots showing the effect of variables for the extraction recovery (%) of Cu(II).

**Figure 4:**
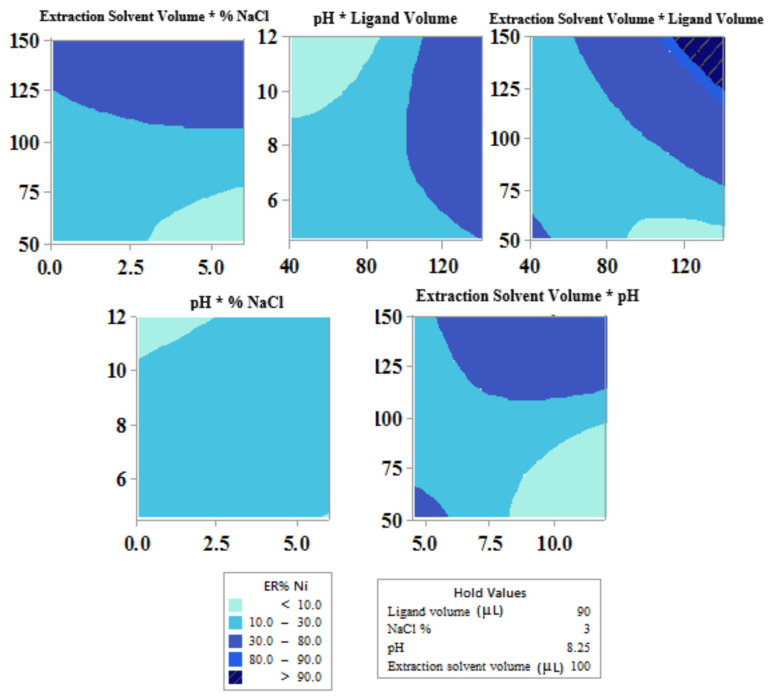
Contour plots showing the effect of variables for the extraction recovery (%) of Ni(II).

The pH has a significant effect in the extraction of Cu(II) and Ni(II) from aqueous solution considering the formation of their complexes. The effect of pH was studied in the range of 2–12 with HCl (1 mol L^−1^), and NaOH (1 mol L^−1^) for pH adjustment. At the low pH values due to the protonated DDTC molecules the ERs of the analytes are low. The ERs in the alkali environment is also reduced as a result of precipitation of the cations. The contour plots of the responses in Figures 3 and 4 are drawn as a function of two factors at a time, holding another factors at the center points. Remarkable interaction of pH with other factors are shown in contour plots in Figures 3 and 4 and confirmed as significant p-vales of interaction coefficients in ANOVA table (Table 4). Results showed that pH in the range of 4–8 had maximum effect on extraction recovery of target analytes.

According to related research, DDTC can react with many types of heavy metals to form complexes [37]. The volume of DDTC (0.05 mol L^−1^) was varied in the range of 40–140 μL. The volume of complex agent influences the ratio of DDTC to the analytes. As shown in contour plots in Figures 3 and 4, higher ligand volume increases the ERs, which may be related to the large ratio of DDTC to the analytes.

The effect of volume of the extraction solvent (1-dodecanol) on ERs of target ions was investigated over the range of 50–150 μL. With regard to the results obtained by ANOVA table (Table 4), the significant interactions are shown in Figures 3 and 4. It is important to note that at extraction volume of <50 μL sediment wasn’t sufficient for analysis. Increasing the 1-dodecanol volume would increase the extracted amount of analytes, whereas further increase leads to dilution.

**Figure 5:**
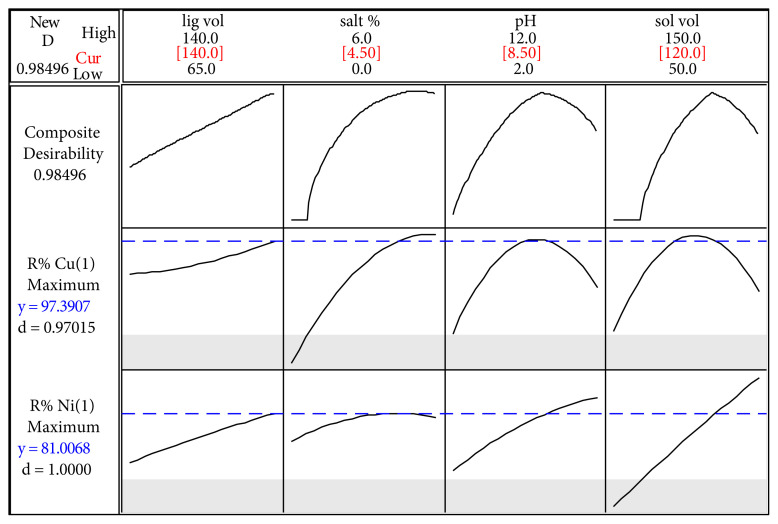
Profiles for predicated values and desirability function for extraction recoveries of Cu(II) and Ni(II).

The salt concentration had noteworthy effect on all responses. The salting-out effect can elucidate the effect of NaCl on the extraction of analytes. The results in Figures 4 and 5 demonstrate the mutual interaction between salt concentration and other factors on extraction of the desired analytes.

#### 3.1.5. Optimization using desirability function

The multi-objective optimization can be approached through the Derringer–Suich desirability function. The maximization of two responses (ER% Cu and ER% Ni) was selected by adjusting the weight or importance. The conditions for the optimization of all desired factors are shown in Figure 5. A weight factor of 1 was preferred for all individual desirability in this study. Applying the methodology of desired function, resulted the following optimum level of parameters, volume of extraction solvent 120 μL, salt 4.5% (w/v), volume of complex agent 140 μL, at pH 8.5, with 97.4% and 81% for ER% Cu and Ni, respectively, and an overall high desirability value of 0.985. A verification test with triplicate was performed under the obtained optimized conditions and the average values for ER% Cu(II) and Ni(II) were 97% and 79%, respectively, with only 0.4% and 2.5% difference between the predicted and experimental extraction recoveries.

### 3.2. Analytical figures of merit

Quantitative parameters of the proposed method, such as the linear range (LR), coefficient of determination (R^2^), limit of detection (LOD), enrichment factor (EF), and extraction recovery (ER), were evaluated under optimum conditions summarized in Table 5. Linear ranges (LR) were 20–100 and 30–200 ng L^−1^ for Cu(II) and Ni (II), respectively. Acceptable linear relationships were found with R^2^ higher than 0.99 in either case. The LODs were calculated by calibration curve method (3S_b_/m, where S_b_ and m are the standard deviation of the blank, and the slope of the calibration curve). Three-time repeatability studies were carried out for EF and ER. EFs were 43 and 32.2 for Cu(II) and Ni(II), respectively, and ERs were in the range of 73%–98%. RSD values were lower than 3.4% for three-time replication indicating that the proposed method has a good repeatability.

### 3.3. Interference effect of other ions

Interference effect of coexisting ions on ERs of the target ions (at 50 ng L^−1^) was examined under optimum conditions described in Section 2.4. Table 6 shows tolerance limits of most commonly occurring interfering ions for both cations (Al^2+^, Zn^2+^, Hg^2+^, Mg^2+^, Ca^2+^) and anions (NO_3_^2−^, Cl^−^, SO_4_^2−^, CO_3_^2−^). The tolerance limit for cations was 100–400 ng L^−1^ and 150–1400 (i.e. higher range for chloride) ng L^−1^ for anions. An ion was considered to be an interfering when it caused a variation greater than ±5 % in the analytical response. The results illustrated that analytes Cu^2+^ and Ni^2+^ at 50 ng L^−1^ were not significantly interfered by coexisting ions. Although Hg^2+^ is not commonly considered as an interfering ion, the reason it was included in this study is that mercury is one the most toxic heavy metals present in aquatic systems, and as such there are great concerns about monitoring the levels of Hg^2+^ and its impact in aqueous media.

### 3.4. Applications of AALLME-SFOD to natural water samples

In this study, the AALLME-SFOD microextraction technique was applied to the analysis of Cu (II) and Ni(II) in a range of natural water samples including surface water, river and well water, summarized in Table 7. Copper was found at a range of 23.30 ± 1.30 (well water) to 40.70 ± 1.40 ng L^−1^ (river water), and nickel at a range of 40.40 + 1.92 (well water) to 44.8 ± 3.30 ng L^−1^ (river water). The river water contained the highest levels of both Cu and Ni and the lowest levels were found in the well waters. The surface water samples resulting mostly from rain showed Cu at 29.40 ± 2.00 and Ni at 41.50 ± 1.60 ng L^−1^.

The accuracy of the method was calculated based on %recovery of the target metal ions from spiked surface, well and river water samples. A standard addition method was applied and the recovery was measured by spiking samples up to 70 ng L^−1^. According to the results presented in Tables 7, Cu(II), and Ni(II) can be quantitatively recovered from the water samples by the applied AALLME-SFOD microextraction method. The %recovery range for copper was from 92 to 96, and for nickel from 86 to 98. As shown in Table 5, the limit of detection (LOD) were 4.5 ng L^−1^ for Ni(II) with a linear range of 30–100 ng L^−1^, and for Cu(II) 10.4 ng L^−1^ at a range of 20–100 ng L^−1^.

### 3.5. Comparison of AALLME-SFOD microextraction method to other methods

Table 8 summarizes several parameters indicative of the analytical performance of the recent published articles involving the determination of metals with microextraction procedure [40–42]. Accordingly, in comparison with solid phase microextraction procedures, the method of this study provides comparable LODs to other methods, high contact area with significant reduction of laboratory equipment, time and cost. Moreover, another advantage is the low RSDs (i.e. <3.4), which could be due to faster equilibration time. Compared to the liquid phase microextractions [29,43–45], the proposed AALLME-SFOD method is exceptional in ultra-trace extraction of the studied analytes and remarkable assay precision. In comparison to LODs suggested by literature [44], the described method offers easier and faster extraction times with simpler laboratory materials.

## 4. Conclusion

Within this work, an AALLME-SFOD was successfully implemented for simultaneous extraction of Cu(II) and Ni(II) from various natural samples (river and well waters in Tabriz, Iran) prior to the GFAAS quantification. The current method is both simpler and faster, as neither time-consuming sample preparation nor expensive equipment are required. The response surface methodology (RMS) as a common tool for optimization in analytical chemistry was used. Multi-criteria approach based on desirability function led to maximum overall desirability D, of 0.985 under optimum condition as presented in Figure 5. A high extraction recovery (ER) of 97.4% and 81% for Cu and Ni, respectively, was obtained with low solvent volume of 120 μL and low volume of complex agent of 140 μL, which support the green chemistry procedure of this optimized AALLME-SFOD. From the analysis of environmental water samples reported in Table 7, copper was found at a range of 23.30 ± 1.30 (well water) to 40.70 ± 1.40 ng L^−1^ (river water), and nickel at a range of 40.40 ± 1.92 (well water) to 44.8 ± 3.30 ng L^−1^ (river water). The river water contained the highest levels of both Cu (II) and Ni (II), and the lowest levels were found in the well water.

The comparison of current study with some previous reported methods (Table 8) for determination of metal ions indicates that this method is comparable in terms of LOD and RSD and has evident advantages as it uses less solvent and can be performed in faster timescales. Estimated values have been evaluated by a verification test and reveal the benefit of utilizing DF methodologies. In general, the application of desirability multi-response approach is promising in analytical research, where it efficiently and objectively selects the best conditions.

## Figures and Tables

**Table 1:** Instrument settings and furnace programs for analysis of Ni(II) and Cu(II) by GFAAS.

Conditions	Cu(II)	Ni(II)

Wavelength (nm)	324.8	232.0

Lamp current (mA)	15	25

Air flow (mL min^−1^)	100, 1000	100, 1000

Injection volume (μL)	10	10

Heating program temperature °C [ramp time (s), hold time (s)]		

Drying 1	150 (20,0)	110 (20, 0)

Drying 2	250 (10,0)	250 (10,0)

Pyrolysis 1	900 (10,0)	1000 (10,0)
Pyrolysis 2	1200 (0,13)	1100 (0,13)

Atomization	2000 (0,2)	2150 (0,2)

Cleaning	2200 (0,2)	2250 (0,2)

**Table 2:** Experimental factors and levels in the central composite design for extraction of Ni(II) and Cu(II) with proposed AALLME-SFOD method.

Independent variables	Code	Variable levels				
		−α	1	0	1	+α
Ligand volume (μL)	X_1_	40	65	90	115	140
Salt % (w/v)	X_2_	0	1.5	3	4.5	6
pH	X_3_	2	4.5	7	9.5	12
Solvent volume (μL)	X_4_	50	75	100	125	150

**Table 3:** Experimental plan and responses for extraction of Ni(II) and Cu(II) with proposed AALLME-SFOD method.

Run	Uncoded value of variables				Experimental results (ER%)	
	X_1_	X_2_	X_3_	X_4_	Cu(II)	Ni(II)
1	90	3	7	100	100.00	21.00
2	65	4.5	4.5	75	45.54	29.50
3	115	1.5	4.5	125	57.24	29.72
4	65	4.5	9.5	125	50.34	29.72
5	90	3	7	50	22.80	22.34
6	115	4.5	9.5	75	83.50	8.50
7	90	3	12	100	12.61*	12.08
8	65	4.5	9.5	75	92.67	5.50
9	65	1.5	9.5	125	30.34	16.21
10	90	0	7	100	72.97	22.00
11	40	3	7	100	43.24*	21.00
12	115	1.5	9.5	75	21.46	19.00
13	65	1.5	9.5	75	67.01	4.00
14	90	3	7	100	87.38	21.00
15	115	4.5	4.5	125	78.62	62.16*
16	115	4.5	9.5	125	85.58	2.19*
17	90	3	7	150	33.05	47.05
18	115	1.5	4.5	75	32.46	25.00
19	115	4.5	4.5	75	21.72	13.00
20	65	1.5	4.5	125	86.20	13.18
21	90	3	7	100	90.09	44.00*
22	90	3	7	100	90.00	61.53*
23	90	6	7	100	54.05	24.17
24	90	3	2	100	53.15	39.56*
25	140	3	7	100	87.83	51.64
26	65	1.5	4.5	75	91.62	47.25
27	90	3	7	100	99.09	33.00
28	65	4.5	4.5	125	51.03	5.40
29	115	1.5	9.5	125	96.55*	78.38*

* Outliers.

**Table 4:** ANOVA and results of regression analysis of uncoded units.

Source	ER% of Cu		ER% of Ni	
	Coefficient	P-value	Coefficient	P-value
Constant	−7.2322	0.93*	395.916	<0.0001
Linear				
(X_1_)	−3.491	0.003	−2.837	<0.0001
(X_2_)	−35.0877	0.02	−14.511	0.086*
(X_3_)	22.4049	0.025	−21.59	0.002
(X_4_)	4.7783	<0.0001	−3.481	<0.0001
Square				
(X_1_*X_1_)	0.0015	0.719*	0.005	0.029
(X_2_*X_2_)	−3.4838	0.002	−0.197	0.691*
(X_3_*X_3_)	−1.7855	0.002	−0.664	0.016
(X_4_*X_4_)	−0.0268	<0.0001	0.004	0.052**
Interaction				
(X_1_*X_2_)	0.2751	0.003	−0.004	0.918*
(X_1_*X_3_)	0.0633	0.164*	0.112	0.002
(X_1_*X_4_)	0.017	0.002	0.015	<0.0001
(X_2_*X_3_)	3.9186	<0.0001	0.939	0.034
(X_2_*X_4_)	0.0536	0.465*	0.09	0.059**
(X_3_*X_4_)	−0.1514	0.004	0.188	<0.0001
R^2^	93.97		95.04	
R^2^_adj_	86.31		86.36	
Lack of fit	0.103		0.882	

Ligand volume (X_1_), salt % (X_2_), pH (X_3_), solvent volume (X_4_).

* Insignificant at “p-value” more than 0.05.

** Coefficient were taken into account in order to the precautionary aspects.

**Table 5:** Quantitative characteristic of the proposed AALLME-SFOD-GFAAS for the analysis of heavy metals.

Analyte	LR^a^ (ng L^−1^)	R^2^^b^	LOD^c^ (ng L^−1^)	RSD%^d^		ER ± SD^e^	EF ± SD^f^
				Intra-day	Inter-days		
Cu(II)	20–100	0.994	10.4	2.3	3.4	97.7 ± 4	43 ± 2
Ni(II)	30–200	0.9973	4.5	2.5	2.8	73.2 ± 4	32.2 ± 3

a Linear range

b Determination coefficient

c Limit of detection

d Relative standard deviation (C = 40 ng L^−1^, n = 6) for intra-day and (C = 40 ng L^−1^, n = 6) for inter-days

e Extraction recovery ± standard deviation (n = 3)

F Enrichment factor ± standard deviation (n = 3).

**Table 6:** Effect of some interference on extraction and determination of 50 ng L^−1^ of Cu(II), and Ni(II).

Species	Tolerance limit of	
	Ni^2+^	Cu^2+^
Al^2+^	100	200
Zn^2+^	100	100
Ca^2+^	250	250
Mg^2+^	400	400
Hg^2+^	100	150
Cl^−^	1200	1200
SO_4_^2−^	150	150
NO_3_^−^	700	1400
CO_3_^−^	375	375

**Table 7:** Results for analysis of environmental water samples and spiked recoveries with proposed method.

Analyte	Spiked (ng L^−1^)	Surface water		Well water		River water	
		Found (ng L^−1^) ± SD (n = 3)	Recovery % ± S.D (n = 3)	Found (ng L^−1^) ± SD (n = 3)	Recovery % ± SD (n = 3)	Found (ng L^−1^) ± SD (n = 3)	Recovery % ± S.D (n = 3)
Cu	0	29.40 ± 2.00	-	23.30 ± 1.30	-	40.70 ± 1.40	-
	40	66.60 + 1.33	93.00 ± 3.00	60.80 ± 1.50	94.00 ± 4.00	78.20 ± 2.20	94.00 ± 6.00
	50	77.02 + 1.19	95.00 ± 2.00	69.90 ± 2.60	93.00 ± 5.00	87.10 ± 2.70	93.00 ± 5.00
	70	97.02 + 3.54	96.00 ± 5.00	90.30 ± 2.60	95.00 ± 4.00	104.00 ± 4.70	92.00 ± 6.00
Ni	0	41.50 ± 1.60	-	40.40 + 1.92	-	44.8 ± 3.30	-
	40	79.80 ± 1.60	95.00 ± 4.00	78.70 ± 2.50	96.00 ± 6.00	84.3 ± 2.50	98.00 ± 6.00
	50	89.80 ± 3.30	96.00 ± 6.00	88.10 ± 2.80	95.00 ± 5.00	94.3 ± 4.10	98.00 ± 8.00
	70	101.50 ± 1.60	86.00 ± 2.00	104.80 ± 4.40	92.00 ± 4.00	109.2 ± 2.50	92.00 ± 4.00

**Table 8:** Comparison of recently published methods and present method.

Analyte	Methods	LR^a^ (μg L^−1^)	LOD b	RSD%^c^	EF^d^	Ref.

Cd	Magnetic-SPE–ICP-OES^e^	1.00–400.00	300.0	1.7–3.2	116–150	42
Co			700.0			
Cr			500.0			
Ni			600.0			
Pb			800.0			
Zn			200.0			

Co	Suspended nanoparticles in surfactant media–(ETAAS)	0.01–0.15	2.5	<6	-	41
Ni		0.01–0.12 0.01–0.25	2.8			
Cu			2.6			

Cu	Membrane-SPME–ICP-OES		880.0	<8	-	40
Mg			610.0			
Ni			380.0			

Co	DLLME–SFOD–GFAAS^f^	5.00–55.00	1.3	7.2	800	30
Ni		5.00–40.00	1.3	7.2	800	

Co	UASEME^g^–GFAAS	0.10–5.00	15.6	4.3	58	44
				7.5		

Co	DLPME^h^–GFAAS	10.00–250.00	21.0	8.2	101	43
Ni			33.0		200	

Co	In situ-CO_2_ disperser-LLME–GFAAS	0.02–0.30	8.0	4.6	148	45
Ni		0.02–0.20	12.0	4.5	139	
Cu		0.015–0.25	6.0	2.3	150	

Cu	AALLME-SFOD-GFAAS	0.02–0.10	4.5	2.8	46	This work
Ni		0.02–0.20	10.4	3.4	46	

a Linear range

b Limit of detection (ng L^−1^)

c Relative standard deviation

d Enrichment factor

e Magnetic solid phase extraction inductively-coupled plasma optical emission spectrometry

F Dispersive liquid–liquid microextraction based on solidification of floating organic drop –graphite furnace atomic absorption spectrometry

g Ultrasound-assisted surfactant-enhanced emulsification microextraction

h Dispersive liquid-phase microextraction.
